# Editorial: Contemporary perspectives in adolescent mental health

**DOI:** 10.3389/fpsyg.2024.1376940

**Published:** 2024-02-12

**Authors:** Alina Cosma, Gina Martin, Sophie D. Walsh, Yekaterina Chzhen, Candace Currie

**Affiliations:** ^1^Department of Sociology, Trinity College Dublin, Dublin, Ireland; ^2^School of Psychology, Trinity College Dublin, Dublin, Ireland; ^3^Faculty of Health Disciplines, Athabasca University, Athabasca, AB, Canada; ^4^Department of Geography and Environment, Western University, London, ON, Canada; ^5^Department of Criminology, Bar Ilan University, Ramat Gan, Israel; ^6^Glasgow Caledonian University London, London, United Kingdom

**Keywords:** gender, stress, depression, social inequalities, COVID-19, parenting

The adolescent years mark a critical phase in human development, shaping the foundation for mental health throughout one's life (Patton et al., 2016). As we delve into the complex landscape of adolescent mental health, it becomes evident that understanding and addressing the unique contemporary challenges faced by this age group are imperative for creating and supporting environments in which adolescents can develop resilience and thrive mentally as well as physically.

Mental health is a cornerstone of young peoples' functioning and increasingly a topic of concern (Cosma et al., 2023). The foundation of mental health begins in childhood but is largely laid in adolescence, and more than half of adult mental health disorders have their onset before the age of 14 (Kessler et al., 2007). Mental health problems are becoming a leading cause of disease burden amongst adolescents globally, affecting their achievement of developmental tasks, socially, personally and academically. Poor adolescent mental health has been linked with poor long-term adult health morbidity and mortality (Patton et al., 2016). Optimal mental health requires a positive sense of wellbeing in addition to the absence of clinically significant, active mental illness. Monitoring systems are key to understanding whether efforts to improve young people's mental health at a national and international level are successful (Viner et al., 2012).

To advance our knowledge, the special topic “*Contemporary perspectives in adolescent mental health*” in *Frontiers in Psychology* provides a platform for cutting-edge research and insights. Taking a contemporary perspective to adolescent mental health means that we recognize that adolescents today are experiencing life in a different context than the generations before; therefore, it is important to continue to update our knowledge of adolescent mental health with this in mind. The last decades have seen great changes in young peoples' lives, including technological advancement, global conflict, environmental change, social polarization, and the COVID-19 pandemic, all which may impact on adolescent mental health (e.g., Martin et al., 2022, 2023; Boer et al., 2023). Accordingly, this collection of papers brings together diverse perspectives including different cultural contexts and research methodologies, shedding light on various facets of adolescent mental health (e.g., time trends, COVID-19 impacts, mechanisms) and offering valuable contributions to this evolving field.

Previous research has outlined the increase in mental health problems in contemporary generations of adolescents (Bor et al., 2014), with increases over time in the age and gender gaps (Cosma et al., 2020; Boer et al., 2023). Our Research Topic includes three papers that advanced knowledge in this area. Eriksson and Stattin used a person-centered approach to identify secular trends in the mental health profiles among 15-year-olds in Sweden. Their findings suggest that the turning point toward decline was 2010, with profiles of psychosomatic complaints registering the highest increase over time. Similarly, in a Scottish sample, covering the same time period and using the same methodology, Inchley et al. reported worsening trends of psychosomatic complaints. However, a widening of the social inequality gap was found, especially for girls. Finally, Li et al. in their focus on sexual minorities showed that the distress is also not shared equally across all adolescent groups, but that we need to be understanding how today's reality may be affecting subgroups of adolescents in unique ways. These studies emphasize a need for nuanced research which acknowledges that the mental health burden is not shared equally across all adolescents and that deeper understanding of causal mechanisms which may explain why certain groups are affected more is needed. For example, Peleg et al. explored the role of emotional distress as a mediator between differentiation of self and the risk of eating disorders among adolescents. Their findings provide valuable insights into the gendered character of these mechanisms.

How adolescent mental health is conceptualized is an important area of inquiry, and is foundational to how this phenomena is understood and studied. In this Research Topic, King et al. explored a continuous measure of adolescent mental health inspired by a dual-factor model of mental health which suggests that mental illness and positive mental health reflect distinct continua, rather than the extreme ends of a single spectrum (Iasiello et al., 2020). This is a novel approach that uses the full range of data available and may be a useful approach for smaller studies with limited statistical power (King et al.). McMahon et al. examined if a measure of COVID-19 anxiety was associated with psychological distress. Their findings indicate that a positive association exists between worries about the indirect consequences of COVID-19 (consequence anxiety) and psychological distress, and that this is moderated by parent-child closeness. Together, these studies provide valuable insights into how we conceptualize adolescent mental health, and both bring insights that can inform future research.

As part of this search for understanding mechanisms explaining adolescent mental health, this Research Topic brings some advancements in understanding contemporary risk factors for developing mental health problems during adolescent years by exploring individual or parent related mechanisms. As such, Yang et al. explored the role of parent-child attachment and emotional insecurity in the association between exposure to interparental conflict and emotional and behavioral problems. Ding et al.'s work investigated the associations between parental phubbing (i.e., parents' concentrating on using cell phones while neglecting their children in parent–child interaction) and adolescent sleep problems and the mediating role of negative emotions and self-control.

In addition, this Research Topic examined how we can reduce the experience of stress among adolescents. In their qualitative investigation, Persson et al. explored school nurses' experiences of health-promoting work to prevent stress in Swedish adolescents. Their results showcase that healthy living habits are seen as a prerequisite for preventing stress. Conversely, in order to maximize the impact of the work it is important for the school nurse to be visible and accessible to create good relationships with the students but also foster collaboration with stakeholders outside the school. Finally, using national longitudinal data, Srivastava et al. examined time-sequential associations between identity management stress and depression over time by sexual identity fluidity. Their results indicate that sexual identity development and fluidity processes differ between cisgender females and males.

The papers featured in this special topic collectively contribute to a nuanced and comprehensive understanding of contemporary perspectives in adolescent mental health. They stress that the mental health burden is not shared equally by all adolescents and that future research needs to focus on an understanding of which adolescents are affected the most and why that may be. More emphasis on causal mechanisms, as well as resilience factors are needed if we are to fully address the decline in adolescent mental health. As we navigate the challenges and opportunities of this critical developmental stage, it is imperative to integrate these insights into policies, practices, and interventions that support the wellbeing of adolescents. By fostering a holistic approach that considers the digital landscape, social inequalities, individual-level mechanisms and cross-cultural differences, we can pave the way for a healthier and more resilient generation.

## Author contributions

AC: Writing – original draft, Writing – review & editing. GM: Writing – original draft, Writing – review & editing. SW: Writing – original draft, Writing – review & editing. YC: Writing – original draft, Writing – review & editing. CC: Writing – original draft, Writing – review & editing.
